# Long-term Outcomes Associated With Open vs Endovascular Abdominal Aortic Aneurysm Repair in a Medicare-Matched Database

**DOI:** 10.1001/jamanetworkopen.2022.12081

**Published:** 2022-05-13

**Authors:** Kevin Yei, Asma Mathlouthi, Isaac Naazie, Nadin Elsayed, Bryan Clary, Mahmoud Malas

**Affiliations:** 1 University of California, San Diego, La Jolla

## Abstract

**Importance:**

Endovascular aneurysm repair is associated with a significant reduction in perioperative mortality and morbidity compared with open aneurysm repair in the treatment of abdominal aortic aneurysm. However, this benefit decreases over time owing to increased reinterventions and late aneurysm rupture after endovascular repair.

**Objective:**

To compare long-term outcomes of endovascular vs open repair of abdominal aortic aneurysm.

**Design, Setting, and Participants:**

This multicenter retrospective cohort study used deidentified data with 6-year follow-up from the Medicare-matched Vascular Quality Initiative Vascular Implant Surveillance and Interventional Outcomes Network database. Patients undergoing first-time elective endovascular or open abdominal aortic aneurysm repair from 2003 to 2018 were propensity score matched. Patients with ruptured abdominal aortic aneurysm, concomitant procedures, or prior history of abdominal aortic aneurysm repair, were excluded. Data were analyzed from January 1, 2003, to December 31, 2018.

**Exposures:**

First-time elective endovascular or open repair for abdominal aortic aneurysm.

**Main Outcomes and Measures:**

The primary long-term outcome of interest was 6-year all-cause mortality, rupture, and reintervention. Secondary outcomes included 30-day mortality and perioperative complications.

**Results:**

Among a total of 32 760 patients (median [IQR] age, 75 [70-80] years; 25 706 [78.5%] men) who underwent surgical abdominal aortic aneurysm repair, 28 281 patients underwent endovascular repair and 4479 patients underwent open repair. After propensity score matching, there were 2852 patients in each group. Open repair was associated with significantly lower 6-year mortality compared with endovascular repair (548 deaths [35.6%] vs 608 deaths [41.2%]; hazard ratio [HR], 0.83; 95% CI, 0.74-0.94; *P* = .002), with increases in mortality starting from 1 to 2 years (84 deaths [4.3%] vs 126 deaths [6.7%]; HR, 0.63; 95% CI, 0.48-0.83; *P* = .001) and 2 to 6 years (211 deaths [25.8%] vs 241 deaths [30.6%]; HR, 0.73; 95% CI, 0.61-0.88; *P* = .001). Open repair, compared with endovascular repair, also was associated with significantly lower rates of 6-year rupture (117 participants [5.8%] vs 149 participants [8.3%]; HR, 0.76; 95% CI, 0.60-0.97; *P* < .001) and reintervention (190 participants [11.6%] vs 267 participants [16.0%]; HR, 0.67; 95% CI, 0.55-0.80; *P* < .001). Open repair was associated with significantly higher odds of 30-day mortality (OR, 3.56; 95% CI, 2.41-5.26; *P* < .001) and complications.

**Conclusions and Relevance:**

These findings suggest that overall mortality after elective abdominal aortic aneurysm repair was higher with endovascular repair than open repair despite reduced 30-day mortality and perioperative morbidity after endovascular repair. Endovascular repair additionally was associated with significantly higher rates of long-term rupture and reintervention. These findings emphasize the importance of careful patient selection and long-term follow-up surveillance for patients who undergo endovascular repair.

## Key Points

**Question:**

What are the long-term outcomes associated with endovascular abdominal aortic aneurysm repair compared with open aneurysm repair?

**Findings:**

In this cohort study of 32 760 abdominal aortic aneurysm repairs in a Medicare-matched database, open repair was associated with higher odds of 30-day mortality and perioperative complications but lower rates of 6-year mortality, rupture, and reintervention.

**Meaning:**

These findings suggest that endovascular abdominal aortic aneurysm repair had perioperative benefits but long-term outcomes remained a significant concern, warranting careful surveillance during long-term follow-up and consideration of open aneurysm repair in appropriate candidates.

## Teaser

This cohort study compares long-term outcomes after endovascular vs open abdominal aortic aneurysm repair.

## Introduction

The use of endovascular repair of abdominal aortic aneurysms (EVAR) has dramatically increased since its first inception in 1991,^1^ accounting for an estimated 74% to 76% of intact abdominal aortic aneurysm (AAA) repairs.^2,3^ The Dutch Randomized Endovascular Aneurysm Management (DREAM),^4^ EVAR-1,^5^ and Open Versus Endovascular Repair (OVER)^6^ multicenter randomized clinical trials all demonstrated decreased perioperative mortality and morbidity with the endovascular approach compared with open aneurysm repair (OAR), which was further confirmed in retrospective studies.^7,8,9,10^

Long-term outcomes after EVAR vs OAR have been more controversial. There was no difference in long-term mortality in 14-years’ follow up of OVER and 15 years’ follow up of DREAM.^11,12^ Similarly, 15-year follow-up in EVAR-1 showed no difference in all-cause mortality, but aneurysm-related mortality was significantly higher in the EVAR group, driven by aneurysm-related mortality starting from 4 years after srugery.^13^

Subsequent observational studies have reinforced the observation of late mortality after EVAR reported in EVAR-1. In 2015, Schermerhorn et al^8^ analyzed Medicare beneficiaries who underwent AAA repair between 2001 and 2008 and were followed up until 2009.^8^ Although there was no difference in overall 8-year mortality, they found a statistically significant increase in mortality after EVAR in the time periods from 60 days to 4 years and from 4 to 8 years.^8^ In a pooled analysis of DREAM, EVAR-1, the study by Schermerhorn et al,^8^ and 4 additional propensity score–matched studies, Takagi et al^14^ found increased mortality with EVAR between 1.8 years and 5 years. Li et al^15^ performed a meta-analysis including 54 studies with 5 to 9 years of follow-up and found significantly higher all-cause mortality after EVAR. Late rupture after EVAR due to graft-related endoleaks has been hypothesized as a possible explanation for these findings.^8,11,13,15,16^

Owing to the concern for late aneurysm rupture and mortality in the context of ever-increasing use of EVAR, these findings warrant further characterization and follow-up. Although randomized trials remain the criterion standard of evidence, they are still subject to many limitations, such as rigorous selection criteria, including comorbidities, and adherence to EVAR device manufacturer instructions for use (IFU); adherence to strict follow-up schedules; and restriction to highly specialized surgeons and centers of care.^17,18^ Retrospective observational studies provide an important adjunct to ensure that results from randomized trials are reflective of outcomes in practice. In this study, we use the Medicare-matched Society for Vascular Surgery (SVS) Vascular Quality Initiative (VQI) Vascular Implant Surveillance and Interventional Outcomes Network (VISION) database to provide the most updated large-scale retrospective analysis on long-term outcomes of elective EVAR and OAR to our knowledge.

## Methods

This cohort study used deidentified information from participating institutions in VQI-VISION; therefore the study was exempt from the need for institutional review board and informed consent. This study is reported following the Strengthening the Reporting of Observational Studies in Epidemiology (STROBE) reporting guideline for observational studies.

This is a retrospective analysis of the prospectively collected SVS VQI-VISION database. The SVS VQI is a well-validated, risk-adjusted data set with robust documentation of demographic, procedural, and postoperative variables from more than 800 hospitals in the United States and Canada.^19^ Variables are extracted from medical records by trained reviewers and quality and accuracy is assessed with robust auditing mechanisms overseen by regional quality groups.

VISION is a partnership between the SVS VQI and Medical Device Epidemiology Network that aims to enhance long-term outcome variables through linkage of SVS VQI data to Medicare claims.^20^ The database accomplishes this through the use of a validated matching algorithm incorporating *Current Procedure Terminology*, *International Classification of Diseases, Ninth Revision (ICD-9)*, and *International Statistical Classification of Diseases and Related Health Problems, Tenth Revision (ICD-10)* codes.^21^

All patients undergoing first-time intact AAA repair between January 1, 2003, and December 31, 2018, were included in this analysis. Patients were divided into 2 groups based on the modality of AAA repair: EVAR and OAR. Patients with ruptured AAA, concomitant procedures, or prior history of AAA repair were excluded (eFigure 1 in the Supplement).

Baseline characteristics compared between groups included year of repair, age, sex, race, ethnicity, body mass index, smoking, diabetes, hypertension, coronary artery disease, congestive heart failure, chronic obstructive pulmonary disease, chronic kidney disease, dialysis, family history of AAA repair, prior coronary artery bypass grafting or percutaneous coronary intervention, prior carotid endarterectomy or carotid artery stenting, prior lower limb revascularization, maximum aortic diameter, symptomatic presentation, and preoperative medication usage.

Race and ethnicity were determined by the VQI race variable, which was extracted from medical records by trained reviewers. Race and ethnicity were assessed because they may be associated with variable outcomes after abdominal aortic aneurysm repair. Year of surgery was divided into 3 categories: 2003 to 2008, 2009 to 2013, and 2014 to 2018. Smoking was divided into 3 categories: never, prior (>1 month before procedure) and current (<1 month before procedure). Hypertension was defined as a documented blood pressure of 130/80 mm Hg or greater on 3 or more occasions. Coronary artery disease was defined as any history of angina or myocardial infarction (MI). Chronic kidney disease was defined as an estimated glomerular filtration rate less than 60 mL/min/1.73 m^2^. An additional sensitivity analysis was performed excluding patients who underwent EVAR who were deemed unfit for OAR on the basis of cardiac status, pulmonary status, frailty, or hostile abdomen.

### Outcomes

Outcomes were compared between EVAR and OAR. The primary outcome of interest was 6-year all-cause mortality, rupture, and reintervention. Secondary outcomes included 30-day mortality and perioperative leg ischemia, intestinal ischemia, MI, respiratory complications, and nonhome discharge. Reintervention was defined as any repeat procedure related to the initial AAA repair or complications after discharge. Leg ischemia was defined as loss of previously palpable pulses, loss of previously present Doppler signals, decrease of greater than 0.15 in ankle-brachial index, development of ischemic rest pain, cyanosis, or tissue loss. Intestinal ischemia was defined as colonoscopy evidence of ischemia, bloody stools with death prior to colonoscopy or laparotomy, or other documented clinical diagnosis. Respiratory complications were defined as pneumonia or ventilator requirement after extubation.

### Statistical Analysis

Categorical baseline characteristics were compared using Pearson χ^2^ test; continuous variables were compared using 2-sample rank-sum tests. Given significant variation in baseline characteristics between groups, we used propensity score matching to compare patients based on procedure type. One-to-one propensity score matching without replacement was used to balance patients on 21 dimensions by the nearest-neighbor principle with a caliper size of 0.2. Matching was performed based on all baseline characteristics to produce a matched cohort of 2842 patients in the EVAR cohort and 2842 patients in the OAR cohort. An adequate match was achieved with an absolute standardized difference less than 0.10 in all baseline covariates, indicating no need for double adjustment.^22^

Pearson χ^2^ test and univariate logistic regression were used to compare perioperative outcomes. Kaplan-Meier analysis and unadjusted Cox proportional hazards regression were used to compare long-term outcomes. Schoenfield residuals and log-log plots were used to assess the proportional-hazards assumption. The proportional hazards assumption was not met for 6-year mortality; thus, this variable was divided into 0- to 1-year, 1- to 2-year, and 2- to 6-year time intervals based on the log-log plot (eFigure 2 in the Supplement).

Patients were stratified by year of repair to analyze temporal trends. Kaplan-Meier analysis, log-rank tests and pairwise Cox proportional hazards regression were used to compare long-term outcomes. A Bonferroni correction was applied to all pairwise comparisons (eTable 2 and eTable 3 in the Supplement).

All analyses were completed using Stata SE version 16.1 (StataCorp). *P* < .05 was considered statistically significant, and tests were 2-sided. Complete case analysis was used to handle missing data (eTable 1 in the Supplement). All values of fewer than 11 were censored in accordance with Centers for Medicare and Medicaid Services cell suppression policy. Data were analyzed from January 1, 2003, to December 31, 2018.

## Results

A total of 32 760 patients (median [IQR] age, 75 [70-80] years; 25 706 [78.5%] men) were included in this analysis, of which 28 281 (86.3%) underwent EVAR and 4479 (13.7%) underwent OAR. In the EVAR group, the median (IQR) age was 75 (70-81) years with 22 588 (79.9%) men. In the OAR group, the median (IQR) age was 72 (68-77) years with 3118 (69.6%) men. There were significant differences in baseline characteristics between groups (Table 1).

**Table 1.** Demographic and Clinical Characteristics of the Sample Before and After Propensity Score Matching

Characteristic	Unmatched (N = 32 760)			Matched (n = 5684)		
	No. (%)		Standardized difference	No. (%)		Standardized difference
	EVAR (n = 28 281)	OAR (n = 4479)		EVAR (n = 2842)	OAR (n = 2842)	
Surgery year						
2003-2008	726 (2.6)	660 (14.7)	0.443	0	0	NA
2009-2013	5401 (19.1)	1204 (26.9)	0.186	540 (19.0)	509 (17.9)	0.028
2014-2018	22 154 (78.3)	2615 (58.4)	0.439	2302 (81.0)	2333 (82.1)	0.028
Age, median (IQR)	75 (70-81)	72 (68-77)	0.415	72 (67-77)	72 (68-77)	−0.041
Sex						
Men	22 588 (79.9)	3118 (69.6)	0.238	1949 (68.6)	1985 (69.8)	0.027
Women	5691 (20.1)	1361 (30.4)	0.238	893 (31.4)	857 (30.2)	0.027
Race						
White	25 920 (91.7)	4184 (93.5)	0.068	2617 (92.1)	2625 (92.4)	0.011
Black	1310 (4.6)	162 (3.6)	0.051	135 (4.8)	122 (4.3)	0.022
Other^a^	1034 (3.7)	130 (2.9)	0.042	90 (3.2)	95 (3.3)	0.010
Hispanic ethnicity	777 (2.8)	86 (1.9)	0.055	79 (2.8)	70 (2.5)	0.019
BMI, median (IQR)	27 (24-31)	26 (23-30)	0.009	27 (23-30)	27 (23-30	0.008
Smoking						
Never	4048 (14.3)	389 (8.7)	0.177	228 (8.0)	239 (8.4)	0.014
Prior	16 284 (57.7)	2279 (51.0)	0.135	1455 (51.2)	1434 (50.5)	0.015
Current	7899 (28.0)	1803 (40.3)	0.263	1159 (40.8)	1169 (41.1)	0.007
Comorbidities						
Diabetes	5850 (20.7)	770 (17.2)	0.089	521 (18.3)	508 (17.9)	0.012
Hypertension	23 389 (83.4)	3793 (84.9)	0.041	2411 (84.8)	2414 (84.9)	0.003
CAD	12 193 (43.2)	1388 (31.1)	0.253	894 (31.5)	809 (28.5)	0.065
CHF	3728 (13.2)	385 (8.6)	0.148	282 (9.9)	264 (9.3)	0.021
COPD	9663 (34.2)	1614 (36.1)	0.039	1095 (38.5)	1030 (36.2)	0.048
CKD	10 495 (37.2)	1646 (36.8)	0.008	1036 (36.5)	1023 (36.0)	0.010
Dialysis	311 (1.1)	27 (0.6)	0.054	22 (0.8)	17 (0.6)	0.021
Family history of AAA repair	2242 (8.0)	495 (11.3)	0.113	285 (10.0)	281 (9.9)	0.005
Prior CABG or PCI	9909 (35.1)	1390 (31.1)	0.085	814 (28.6)	795 (28.0)	0.015
Prior CEA or CAS	1446 (5.7)	199 (6.1)	0.017	170 (6.0)	165 (5.8)	0.007
Prior lower limb revascularization	1912 (6.8)	341 (7.6)	0.033	217 (7.6)	210 (7.4)	0.009
Maximum aortic diameter, median (IQR), mm	55 (52-60)	58 (53-66)	−0.365	57 (52-67)	57 (53-65)	0.066
Symptomatic presentation	2302 (8.2)	561 (12.6)	0.145	406 (14.3)	355 (12.5)	0.053
Preoperative medications						
ACE inhibitors	11 506 (45.5)	1447 (44.7)	0.017	1234 (43.4)	1273 (44.8)	0.028
Anticoagulant	3294 (13.0)	283 (8.7)	0.138	246 (8.7)	257 (9.0)	0.014
P2Y12 inhibitors	3550 (12.6)	377 (8.4)	0.135	274 (9.6)	259 (9.1)	0.018
Aspirin	18 642 (66.0)	2994 (67.0)	0.020	1795 (63.2)	1837 (64.6)	0.031
β-blocker	15 671 (55.5)	2900 (64.9)	0.192	1662 (58.5)	1638 (57.6)	0.017
Statin	19 953 (70.7)	3138 (70.2)	0.011	2057 (72.4)	2037 (71.7)	0.016

Abbreviations: AAA, abdominal aortic aneurysm; ACE, angiotensin-converting enzyme; BMI, body mass index (calculated as weight in kilograms divided by height in meters squared); CABG, coronary artery bypass grafting; CAD, coronary artery disease; CAS, carotid artery stenting; CEA, carotid endarterectomy; CHF, congestive heart failure; CKD, chronic kidney disease; COPD, chronic obstructive pulmonary disease; EVAR, endovascular aneurysm repair; OAR, open aneurysm repair; PCI, percutaneous coronary intervention.

^a^ Documented as American Indian or Alaskan Native, Asian, Native Hawaiian or other Pacific Islander, or unknown or other according to Vascular Quality Initiative race variable.

After propensity score matching, there were 2852 patients in each group. In the EVAR group, the median (IQR) age was 72 (67-77) years with 1949 (68.6%) men. In the OAR group, the median (IQR) age was 72 (68-77) years with 1985 (69.8%) men. There were no residual baseline imbalances with standardized difference greater than 0.10 (Table 1).

### Long-term Outcomes

Over 6 years in the unmatched cohort, patients who underwent OAR, compared with those who underwent EVAR, had significantly lower rates of mortality (1058 deaths [34.9%] vs 6348 deaths [43.5%]; *P* < .001), rupture (197 patients [5.6%]) vs 1385 patients [7.7%]; *P* < .001), and reintervention (329 patients [9.8%] vs 2619 patients [15.3%]; *P* < .001) (Table 2; eFigure 3 in the Supplement). After dividing 6-year mortality into shorter time intervals, the OAR group, compared with the EVAR group, had significantly lower rates of mortality from 1 to 2 years (141 deaths [4.1%] vs 1404 deaths [7.5%; *P* < .001) and 2 to 6 years (417 deaths [17.8%] vs 2816 deaths [24.5%]; *P* < .001). OAR was associated with a statistically significant increase in mortality at 1 year (389 deaths [9.0%] vs 2128 deaths [8.2%]; *P* = .02).

**Table 2.** Long-term Outcomes After Open or Endovascular Abdominal Aortic Aneurysm Repair

Outcome	Unmatched (n = 32 760)				Matched (n = 5684)			
	No. (%)		HR (95% CI)^a^	*P* value	No. (%)		HR (95% CI)^a^	*P* value
	EVAR (n = 28 281)	OAR (n = 4479)			EVAR (n = 2842)	OAR (n = 2842)		
Death								
6 y	6348 (43.5)	1058 (34.9)	0.79 (0.74-0.84)	<.001	608 (41.2)	548 (35.6)	0.83 (0.74-0.94)	.002
1 y	2128 (8.2)	389 (9.0)	1.14 (1.02-1.27)	.02	241 (9.2)	253 (9.3)	1.06 (0.89-1.27)	.49
1-2 y	1404 (7.5)	141 (4.1)	0.55 (0.46-0.65)	<.001	126 (6.7)	84 (4.3)	0.63 (0.48-0.83)	.001
2-6 y	2816 (24.5)	417 (17.8)	0.69 (0.63-0.77)	<.001	241 (30.6)	211 (25.8)	0.73 (0.61-0.88)	.001
Rupture, 6 y	1385 (7.7)	197 (5.6)	0.76 (0.65-0.88)	.001	149 (8.3)	117 (5.8)	0.76 (0.60-0.97)	.03
Reintervention, 6 y	2619 (15.3)	329 (9.8)	0.64 (0.57-0.72)	<.001	267 (16.0)	190 (11.6)	0.67 (0.55-0.80)	<.001

Abbreviations: EVAR, endovascular aneurysm repair of abdominal aortic aneurysms; HR, hazard ratio; OAR, open aneurysm repair.

^a^ Using EVAR as the reference group.

**Figure 1.**
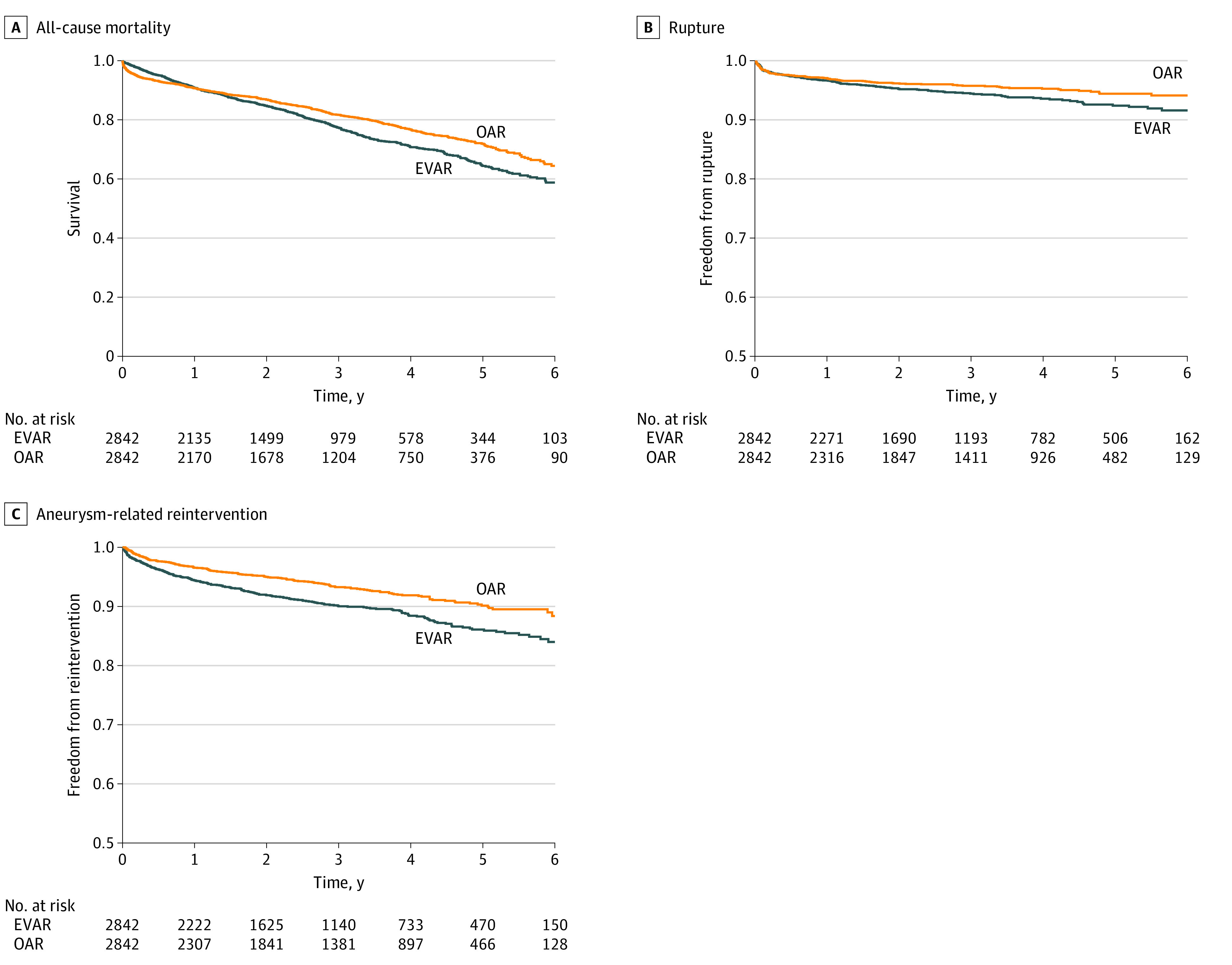
Propensity-Matched 6-Year Mortality, Rupture, and Reintervention After Abdominal Aortic Aneurysm Repair EVAR indicates endovascular aneurysm repair; OAR, open aneurysm repair.

After propensity score matching, patients who underwent OAR, compared with those who underwent EVAR, still had significantly lower rates of mortality (548 deaths [35.6%] vs 608 deaths [41.2%]; HR, 0.83; 95% CI, 0.74-0.94; *P* = .002), rupture (117 patients [5.8%] vs 149 patients [8.3%]; HR, 0.76; 95% CI, 0.60-0.97; *P* = .03), and reintervention (190 patients [11.6%] vs 267 patients [16.0%]; HR, 0.67; 95% CI, 0.55-0.80; *P* < .001) (Table 2; Figure 1). After dividing 6-year mortality into shorter time intervals, OAR, compared with EVAR, was associated with significantly lower rates of mortality from 1 to 2 years (84 deaths [4.3%] vs 126 deaths [6.7%]; HR, 0.63; 95% CI, 0.48-0.83; *P* = .001) and 2 to 6 years (211 deaths [25.8%] vs 241 deaths [30.6%]; HR, 0.73; 95% CI, 0.61-0.88; *P* = .001). There was no difference between OAR and EVAR in mortality at 1 year (253 deaths [9.3%] vs 241 deaths [9.2%]; HR, 1.06; 95% CI, 0.89-1.27; *P* = .49) (Table 2; eFigure 2 in the Supplement).

**Figure 2.**
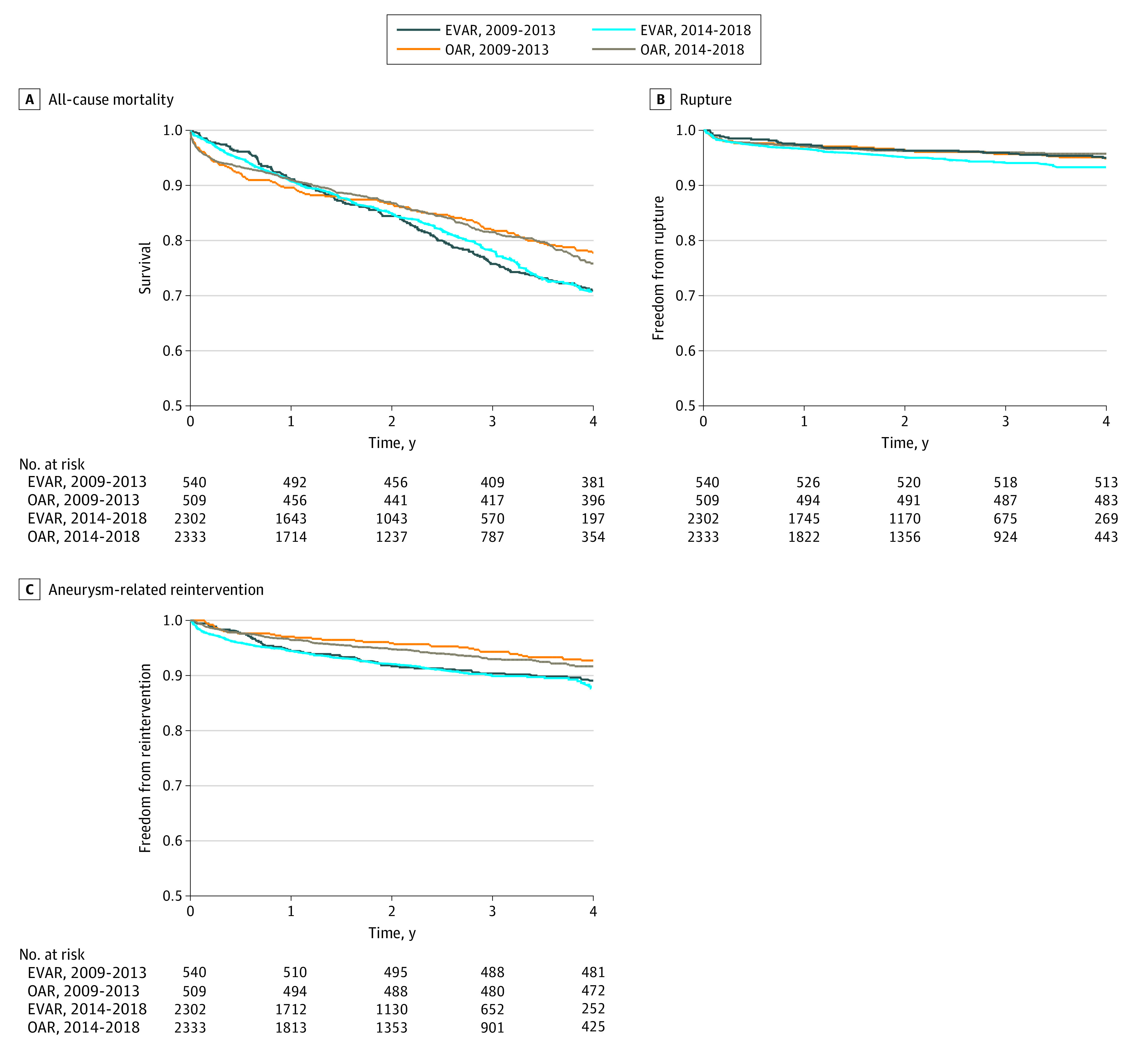
Temporal Trends in Mortality, Rupture, and Reintervention After Abdominal Aortic Aneurysm Repair EVAR indicates endovascular aneurysm repair; OAR, open aneurysm repair.

In stratified analysis dividing patients who underwent EVAR or OAR based on the year in which initial repair was performed, patients who underwent EVAR between 2014 and 2018 had significantly higher rates of reintervention compared with patients who underwent OAR between 2009 and 2013 (185 patients [11.8%] vs 37 patients [7.3%]; HR, 1.72; 95% CI, 1.06-2.78; *P* = .02) and patients who underwent OAR between 2014 and 2018 (135 patients [8.4%]; HR, 1.52; 95% CI, 1.12-2.04; *P* = .002). There were no statistically significant differences in all other pairwise comparisons (Figure 2; eTable 2 and eTable 3 in the Supplement).

In our sensitivity analysis excluding patients who underwent EVAR because they were deemed unfit for OAR, a total of 4037 patients (14.5%) were excluded. Propensity matching produced a balanced cohort of 2833 patients in each group. The OAR group, compared with the EVAR group, had significantly lower rates of rupture (117 patients [5.8%] vs 145 patients [8.1%]; HR, 0.77; 95% CI, 0.60-0.98; *P* = .04) and reintervention (190 patients [11.7%] vs 242 patients [15.2%]; HR, 0.72; 95% CI, 0.59-0.87; *P* = .001), but not mortality (544 deaths [35.3%] vs 534 deaths [38.7%]; HR, 0.94; 95% CI, 0.84-1.06; *P* = .33). However, after dividing mortality into shorter time intervals, the OAR group, compared with the EVAR group, had significantly lower mortality between 2 and 6 years (209 deaths [25.5%] vs 219 patients [29.3%]; HR, 0.81; 95% CI, 0.67-0.98; *P* = .03) (eTable 4 in the Supplement).

### Perioperative Outcomes

After propensity score matching, patients who underwent OAR had significantly higher 30-day mortality compared with those who underwent EVAR (114 deaths [4.0%] vs 33 deaths [1.2%]; odds ratio [OR], 3.56; 95% CI, 2.41-5.26; *P* < .001). They also had higher rates of perioperative complications, including leg ischemia (OR, 2.35; 95% CI, 1.47-3.76; *P* < .001), intestinal ischemia (OR, 12.60; 95% CI, 6.60-24.06; *P* < .001), MI (OR, 6.48; 95% CI, 3.07-13.67; *P* < .001), respiratory complications (OR, 5.82; 95% CI, 4.36-7.76; *P* < .001), and nonhome discharge (OR, 5.10; 95% CI, 4.33-6.00; *P* < .001) (Table 3).

**Table 3.** Perioperative Outcomes After Open or Endovascular Abdominal Aortic Aneurysm Repair

Outcome	Unmatched (n = 32 760)				Matched (n = 5684)			
	No. (%)		OR (95% CI)^a^	*P* value	No. (%)		OR (95% CI)^a^	*P* value
	EVAR (n = 28 281)	OAR (n = 4479)			EVAR (n = 2842)	OAR (n = 2842)		
Death	352 (1.2)	168 (3.8)	3.09 (2.57-3.73)	<.001	33 (1.2)	114 (4.0)	3.56 (2.41-5.26)	<.001
Leg ischemia	218 (0.8)	86 (1.9)	2.52 (1.96-3.24)	<.001	25 (0.9)	58 (2.0)	2.35 (1.47-3.76)	<.001
Intestinal ischemia	111 (0.4)	181 (4.1)	10.70 (8.43-13.58)	<.001	10 (0.4)	121 (4.3)	12.60 (6.60-24.06)	<.001
MI	104 (0.4)	99 (2.2)	6.13 (4.65-8.09)	<.001	NA^b^	NA^b^	6.48 (3.07-13.67)	<.001
Respiratory	396 (1.4)	502 (11.2)	8.90 (7.77-10.19)	<.001	57 (2.0	302 (10.6)	5.82 (4.36-7.76)	<.001
Nonhome discharge	2048 (7.2)	1247 (27.9)	4.95 (4.57-5.35)	<.001	207 (7.3)	813 (28.6)	5.10 (4.33-6.00)	<.001

Abbreviations: EVAR, endovascular aneurysm repair; MI, myocardial infarction; NA, not applicable; OAR, open aneurysm repair; OR, odds ratio.

^a^ Using EVAR as the reference group.

^b^ Censored in accordance with CMS cell suppression policy.

## Discussion

In this cohort study using the Medicare-matched VQI-VISION database, we found significant disparities in long-term outcomes of patients who underwent OAR vs EVAR for first-time elective AAA repair. At 6 years, OAR was associated with significant advantages compared with EVAR, including associations with a 17% reduction in the risk of mortality, a 24% reduction in the risk of rupture, and a 33% reduction in the risk of reintervention.

The 30-day mortality rate observed in our study was 1.2% after EVAR and 3.8% after OAR. These values lie within the range of perioperative mortality observed in randomized trials,^4,5,6^ as well as large-scale retrospective studies.^7,8,9,10^ We also observed fewer perioperative complications associated with EVAR, consistent with the overall consensus of lower perioperative morbidity and mortality associated with elective EVAR compared with OAR in the general population.

For long-term outcomes, our findings suggest that the early mortality benefit associated with EVAR was lost after 1 to 2 years, which is consistent with the findings of DREAM and EVAR-1.^12,13^ However, we also found increased overall mortality over 6 years. This discrepancy between long-term mortality in multiple retrospective observational studies, including ours, and randomized clinical trials warrants careful investigation and consideration of factors that may potentially explain this finding.

Late rupture has previously been hypothesized to be an important cause of long-term mortality in the EVAR population. Numerous studies have demonstrated increased risk of late rupture after EVAR.^8,11,13,15^ Consistent with this, we found a higher rate of rupture after EVAR that persisted through 6 years of follow-up. The main cause of late rupture is thought to be delayed type II endoleak first detected more than 1 year after intervention, which has demonstrated worse outcomes compared with early endoleak.^16,23,24^ Additional contributors to late rupture include undiagnosed type I or III endoleak and graft migration or infection.^25,26^ Late rupture remains a devastating complication, with an estimated 30-day mortality of 32%, according to a 2015 meta-analysis by Antoniou et al.^16^

Prevention of late rupture may entail reintervention for graft-related complications, such as endoleak or migration, resulting in higher rates of reintervention after EVAR compared with OAR.^8,27^ Although midterm results of DREAM, EVAR-1, and OVER showed conflicting results regarding reintervention, the most recent reports of these trials that included both EVAR aneurysm-related reinterventions and OAR abdominal wall operations all demonstrate higher reintervention rates after EVAR (DREAM: EVAR, 37.8%; OAR, 21.1%; EVAR-1: EVAR, 26%; OAR 12%; OVER: EVAR, 26.7%; OAR, 19.8%).^11,12,13^ In line with these studies, we also found a higher rate of reintervention after EVAR that persisted through 6 years of follow-up. However, our results are limited by the absence of incisional hernia repair and other abdominal wall operations in the VQI-VISION database, likely resulting in an underestimate of reinterventions in the OAR group. Regardless, reintervention after EVAR remains a significant concern. A 2021 study by Columbo et al^28^ of Medicare-matched VQI patients reported that nearly two-thirds of reinterventions after EVAR required a hospital stay of 3 or more days, with the most significant risk factors associated with reintervention being emergent intervention, aneurysm size greater than 6.0 cm, prior aortic surgical treatment, iliac artery aneurysm, and procedure time. Careful surveillance in patients with these characteristics is particularly important, as 20% to 40% of patients who undergo EVAR may be lost to follow-up with increased morbidity and mortality.^29,30^

Significant advancements have been made in EVAR technology over the years, including graft design, deployment techniques, and imaging modalities.^31,32^ To investigate the potential associations of these advancements with our outcomes, we performed a stratified analysis by year of repair. Pairwise comparison of these groups showed no differences aside from higher rates of reintervention after EVAR performed between 2014 and 2018 compared with OAR performed in either time group (2009-2013 or 2014-2018). In particular, there was no difference in mortality, rupture, or reintervention with EVAR performed between 2014 and 2018 compared with EVAR performed between 2009 and 2013. However, there was also no difference in mortality and rupture with OAR compared with EVAR, which may indicate loss of power owing to reduced sample size on further stratification of our propensity-matched cohort.

We additionally performed a sensitivity analysis excluding patients who underwent EVAR who were not candidates for OAR. Although there was no difference in 6-year survival, 1-year survival, or 1- to 2-year survival, OAR remained associated with greater overall survival after 2 years, as well as lower risk of rupture or reintervention over 6 years. These findings suggest that although EVAR remains a valuable option in patients ineligible for OAR, as demonstrated in the EVAR-2 trial,^33^ careful weighing of risks and benefits is warranted in patients who are candidates for either repair modality.

### Limitations

This study has some limitations. Several factors that exist outside of our current database may contribute to mortality after elective AAA repair. One such factor is the use of EVAR devices outside manufacture IFU. Randomized trials, such as DREAM,^4^ EVAR-1,^5^ and OVER,^6^ were performed only in patients meeting IFU criteria and did not find differences in long-term all-cause mortality. However, retrospective studies indicate that nearly half of patients currently being treated with EVAR may have 1 or more features outside IFU, with higher rates of graft-related adverse events and aneurysm-related mortality.^34,35,36^ Thus off-label EVAR use may be an important contributor to long-term mortality, reintervention, and rupture in clinical outcomes that we are unable to account for in this database.

Another factor to consider is adherence to long-term surveillance recommendations, as this represents another potential gap between randomized trials and clinical practice. Although VISION provides robust data on long-term outcomes via Medicare linkage, it does not indicate loss to follow-up surveillance, which may contribute to worse survival after EVAR.^30^ However, further investigation is needed in this regard, as other studies have demonstrated no survival benefit with adherence to surveillance recommendations.^37,38^

In addition, we are only able to assess overall mortality and not aneurysm-related mortality. Since our study included only Medicare patients, our results may not be generalizable to younger patients who have been shown to have more comparable perioperative outcomes after elective EVAR and OAR.^39^ We were also unable to assess hospital-level factors, such as case volume and practice patterns, which have been associated with elective AAA repair outcomes.^40^

Further limitations of our study include those shared by all large database studies, including underreporting, coding errors, and missing data. However, the SVS VQI is a well-audited data set with rigorous quality control measures that seek to limit such errors.^19^ Although we performed propensity score matching to minimize the confounding of differences in baseline demographic and operative characteristics, some degree of confounding by indication or selection bias and unmeasured variables is unavoidable with this study design.

## Conclusions

In this large-scale cohort study, overall mortality after elective AAA repair was higher with EVAR than OAR over 6 years, despite reduced 30-day mortality and perioperative morbidity after EVAR. After dividing the mortality analysis into finer time intervals, the mortality benefit associated with EVAR was lost after 1 to 2 years. EVAR additionally demonstrated significantly higher rates of 6-year rupture and reintervention. Although EVAR remains a highly valuable treatment modality, especially in patients who are not candidates for open surgery, OAR should be carefully considered in patients who may be poor candidates for EVAR. Adherence to IFU and long-term follow-up recommendations represent 2 key factors that lie outside the scope of the SVS VQI database and warrant further investigation with respect to long-term outcomes.

## Notes

Supplement. **eTable 1.** Missing Demographic and Outcome Variables **eTable 2.** Log-Rank Tests for Temporal Trends in Long-term Outcomes After Open or Endovascular AAA Repair **eTable 3.** Pairwise Cox Regression for Temporal Trends in Long-term Outcomes After Open or Endovascular AAA Repair **eTable 4.** Long-term Outcomes after Open or Endovascular AAA Repair in Patients Without Contraindications for Open Repair **eFigure 1.** Flowchart of Cohort Creation **eFigure 2.** Propensity-Matched Time-Divided Mortality After AAA Repair **eFigure 3.** Unmatched Mortality, Rupture, and Reintervention After AAA Repair
