# Supplementary material for: Long-term Outcomes Associated With Open vs Endovascular Abdominal Aortic Aneurysm Repair in a Medicare-Matched Database
Source: JAMA Netw Open. 2022 May 13;5(5):e2212081. doi: 10.1001/jamanetworkopen.2022.12081 (PMC9107027; doi:10.1001/jamanetworkopen.2022.12081)
Supplement: Supplement. — eTable 1. Missing Demographic and Outcome Variables eTable 2. Log-Rank Tests for Temporal Trends in Long-term Outcomes After Open or Endovascular AAA Repair eTable 3. Pairwise Cox Regression for Temporal Trends in Long-term Outcomes After Open or Endovascular AAA Repair eTable 4. Long-term Outcomes after Open or Endovascular AAA Repair in Patients Without Contraindications for Open Repair eFigure 1. Flowchart of Cohort Creation eFigure 2. Propensity-Matched Time-Divided Mortality After AAA Repair eFigure 3. Unmatched Mortality, Rupture, and Reintervention After AAA Repair [file jamanetwopen-e2212081-s001.pdf]

Supplemental Online Content

Yei K, Mathlouthi A, Naazie I, Elsayed N, Clary B, Malas M. Long-term outcomes associated with open vs endovascular abdominal aortic aneurysm repair in a Medicare-matched database. *JAMA Netw Open*. 2022;5(5):e2212081. doi:10.1001/jamanetworkopen.2022.12081

eTable 1. Missing Demographic and Outcome Variables

eTable 2. Log-Rank Tests for Temporal Trends in Long-term Outcomes After Open or Endovascular AAA Repair

eTable 3. Pairwise Cox Regression for Temporal Trends in Long-term Outcomes After Open or Endovascular AAA Repair

eTable 4. Long-term Outcomes after Open or Endovascular AAA Repair in Patients Without Contraindications for Open Repair

eFigure 1. Flowchart of Cohort Creation

eFigure 2. Propensity-Matched Time-Divided Mortality After AAA Repair

eFigure 3. Unmatched Mortality, Rupture, and Reintervention After AAA Repair

This supplemental material has been provided by the authors to give readers additional information about their work.

© 2022 Yei K et al. *JAMA Network Open.*

eTable 1. Missing Demographic and Outcome Variables

	Unmatched (N=32,760)		Matched (N=5,684)	
	**EVAR (n=28,281, 86.3%)**	**OAR (n=4,479, 13.7%)**	**EVAR (n=2,842, 50%)**	**OAR (n=2,842, 50%)**
Surgery Year	0 (0%)	0 (0%)	0 (0%)	0 (0%)
Age, median (IQR)	0 (0%)	0 (0%)	0 (0%)	0 (0%)
Female Sex	N/A** (<0.1%)	0 (0%)	0 (0%)	0 (0%)
Race	17 (0.1%)	N/A** (<0.1%)	0 (0%)	0 (0%)
Hispanic Ethnicity	101 (0.4%)	29 (<0.1%)	N/A** (<0.1%)	N/A** (<0.1%)
BMI, median (IQR)	111 (0.4%)	59 (<0.1%)	0 (0%)	0 (0%)
Smoking	50 (0.2%)	N/A** (<0.1%)	0 (0%)	0 (0%)
Diabetes	54 (0.2%)	11 (<0.1%)	0 (0%)	0 (0%)
HTN	227 (0.8%)	N/A** (<0.1%)	0 (0%)	0 (0%)
CAD	51 (0.2%)	12 (<0.1%)	0 (0%)	0 (0%)
CHF	57 (0.2%)	N/A** (<0.1%)	0 (0%)	0 (0%)
COPD	55 (0.2%)	N/A** (<0.1%)	N/A** (<0.1%)	0 (0%)
CKD	48 (0.2%)	N/A** (<0.1%)	0 (0%)	0 (0%)
Dialysis	48 (0.2%)	N/A** (<0.1%)	0 (0%)	0 (0%)
Family history of AAA repair	264 (0.9%)	107 (<0.1%)	0 (0%)	0 (0%)
Prior CABG/PCI	56 (0.2%)	13 (<0.1%)	0 (0%)	0 (0%)
Prior CEA/CAS	3,076 (10.9%)	1,236 (0.3%)	0 (0%)	0 (0%)
Prior lower limb revascularization	61 (0.2%)	N/A** (<0.1%)	0 (0%)	0 (0%)
Maximum Aortic Diameter, mm median (IQR)	1,471 (5.2%)	186 (<0.1%)	0 (0%)	0 (0%)
Symptomatic Presentation	82 (0.3%)	15 (<0.1%)	0 (0%)	0 (0%)
Preoperative ACE inhibitors	3,010 (10.6%)	1,241 (0.3%)	0 (0%)	0 (0%)
Preoperative Anticoagulant	3,013 (10.7%)	1,242 (0.3%)	0 (0%)	0 (0%)
Preoperative P2Y12 Inhibitors	64 (0.2%)	11 (<0.1%)	0 (0%)	0 (0%)
Preoperative Aspirin	54 (0.2%)	N/A** (<0.1%)	0 (0%)	0 (0%)
Preoperative Beta-Blocker	60 (0.2%)	N/A** (<0.1%)	0 (0%)	0 (0%)
Preoperative Statin	62 (0.2%)	N/A** (<0.1%)	0 (0%)	0 (0%)
Death	0 (0%)	15 (<0.1%)	0 (0%)	0 (0%)
Leg Ischemia	63 (0.2%)	14 (<0.1%)	N/A** (<0.1%)	N/A** (<0.1%)
Intestinal Ischemia	64 (0.2%)	15 (<0.1%)	N/A** (<0.1%)	N/A** (<0.1%)
MI	61 (0.2%)	13 (<0.1%)	N/A** (<0.1%)	N/A** (<0.1%)
Respiratory	63 (0.2%)	13 (<0.1%)	N/A** (<0.1%)	N/A** (<0.1%)

© 2022 Yei K et al. *JAMA Network Open.*

Non-home Discharge	13 (<0.1%)	N/A** (<0.1%)	0 (0%)	0 (0%)

** censored in accordance with CMS cell suppression policy

eTable 2. Log-Rank Tests for Temporal Trends in Long-term Outcomes After Open or Endovascular AAA Repair

Outcome	EVAR, 2009-2013 (n=540, 9.5%)	OAR, 2009-2013 (n=509, 9.0%)	EVAR, 2014-2018 (n=2,302, 40.5%)	OAR, 2014-2018 (n=2,333, 41.0%)	P-value
Death	159 (29.4%)	113 (22.2%)	389 (29.6%)	372 (24.2%)	0.02*
Rupture	27 (5.0%)	26 (5.1%)	111 (6.7%)	84 (4.3%)	0.11
Reintervention	59 (10.9%)	37 (7.3%)	185 (11.8%)	135 (8.4%)	<0.001*

*denotes p-value <0.05

eTable 3. Pairwise Cox Regression for Temporal Trends in Long-term Outcomes After Open or Endovascular AAA Repair

	Reference Group			
	**EVAR, 2009-2013 (n=540, 9.5%)**	**OAR, 2009-2013 (n=509, 9.0%)**	**EVAR, 2014-2018 (n=2,302, 40.5%)**	**OAR, 2014-2018 (n=2,333, 41.0%)**
EVAR, 2009-2013		D: 1.35 (0.98-1.89), p=0.08<br>Ru: 0.98 (0.47-2.00), p>0.99<br>Re: 1.54 (0.88-2.70), p=0.24	D: 1.05 (0.82-1.37), p>0.99<br>Ru: 0.76 (0.43-1.35), p>0.99<br>Re: 0.89 (0.60-1.33), p>0.99	D: 1.23 (0.95-1.59), p=0.18<br>Ru: 1.06 (0.59-1.92), p>0.99<br>Re: 1.35 (0.89-2.04), p=0.33
OAR, 2009-2013	D: 0.74 (0.53-1.02), p=0.08<br>Ru: 1.02 (0.50-2.12), p>0.99<br>Re: 0.65 (0.37-1.13), p=0.24		D: 0.78 (0.58-1.04), p=0.13<br>Ru: 0.78 (0.44-1.39), p>0.99<br>Re: 0.58 (0.36-0.94), p=0.02*	D: 0.91 (0.68-1.20), p>0.99<br>Ru: 1.20 (0.60-2.00), p>0.99<br>Re: 0.88 (0.54-1.43), p>0.99
EVAR, 2014-2018	D: 0.95 (0.73-1.22), p>0.99<br>Ru: 1.32 (0.74-2.33), p>0.99<br>Re: 1.12 (0.75-1.67), p>0.99	D: 1.28 (0.96-1.71), p=0.13<br>Ru: 1.28 (0.72-2.29), p>0.99<br>Re: 1.72 (1.06-2.78), p=0.02*		D: 1.16 (0.96-1.41), p=0.23<br>Ru: 1.41 (0.96-2.04), p=0.11<br>Re: 1.52 (1.12-2.04), p=0.002*
OAR, 2014-2018	D: 0.81 (0.63-1.05), p=0.18<br>Ru: 0.94 (0.52-1.69), p>0.99<br>Re: 0.74 (0.49-1.12), p=0.33	D: 1.10 (0.83-1.47), p>0.99<br>Ru: 0.91 (0.50-1.66), p>0.99<br>Re: 1.14 (0.70-1.86), p>0.99	D: 0.86 (0.71-1.04), p=0.23<br>Ru: 0.71 (0.49-1.04), p=0.11<br>Re: 0.66 (0.49-0.89), p=0.002*	

*denotes p-value <0.05

© 2022 Yei K et al. *JAMA Network Open.*

eTable 4. Long-term Outcomes after Open or Endovascular AAA Repair in Patients Without Contraindications for Open Repair

**Outcome**	**EVAR (n=2,833, 50%)**	**OAR (n=2,833, 50%)**	**HR (95%CI) REF=EVAR**	**P-value**
Death				
6 Years	534 (38.7%)	544 (35.3%)	0.94 (0.84-1.06)	0.33
1 Year	213 (8.2%)	250 (0.2%)	1.18 (0.98-1.41)	0.08
1-2 Years	102 (5.7%)	85 (4.3%)	0.77 (0.58-1.03)	0.08
2-6 Years	219 (29.3%)	209 (25.5%)	0.81 (0.67-0.98)	0.03*
Rupture 6 Years	145 (8.1%)	117 (5.8%)	0.77 (0.60-0.98)	0.04*
Reintervention 6 Years	242 (15.2%)	190 (11.7%)	0.72 (0.59-0.87)	0.001*

*denotes p-value <0.05

© 2022 Yei K et al. *JAMA Network Open.*

eFigure 1. Flowchart of Cohort Creation

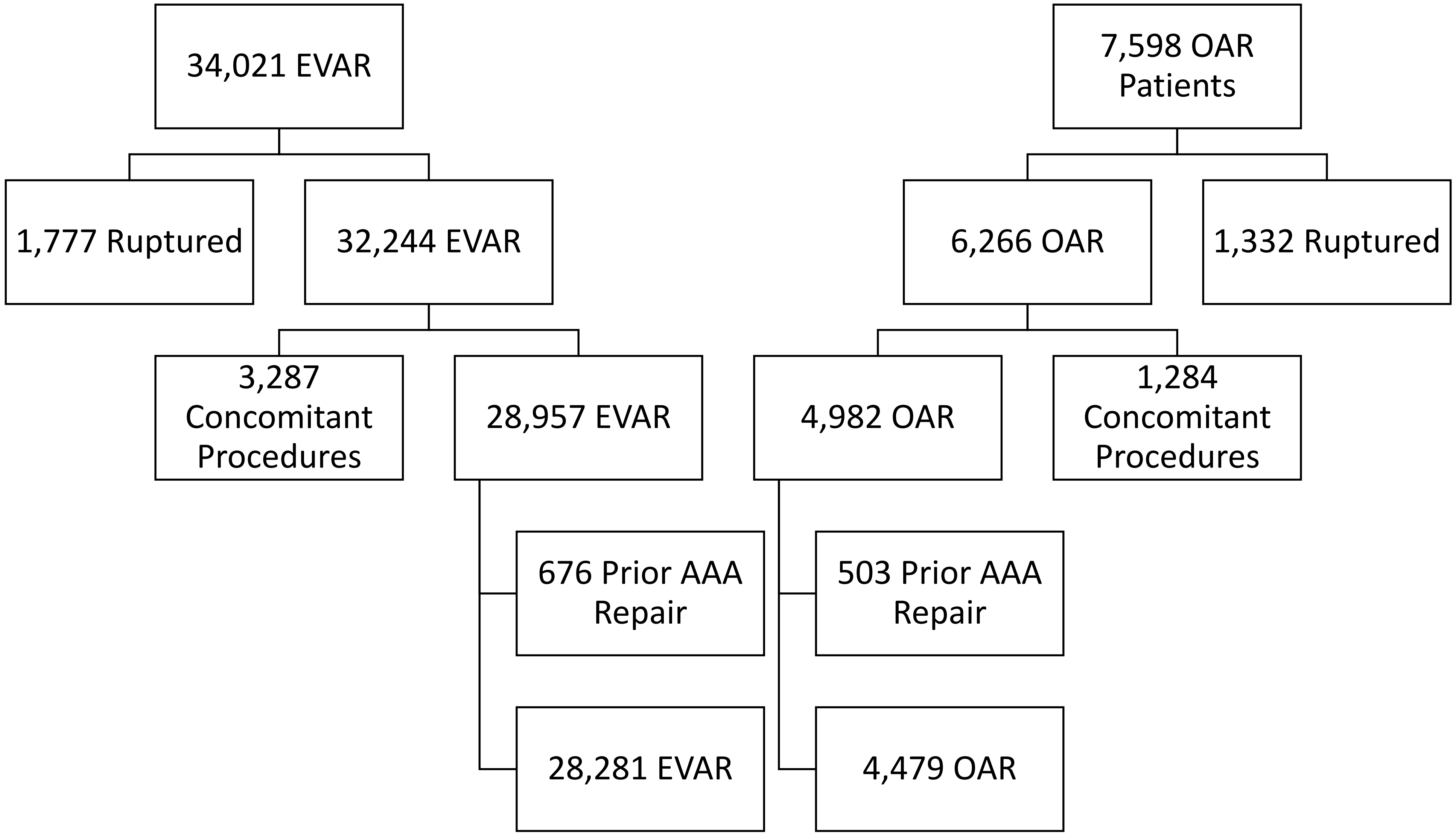

© 2022 Yei K et al. *JAMA Network Open.*

eFigure 2. Propensity-Matched Time-Divided Mortality After AAA Repair

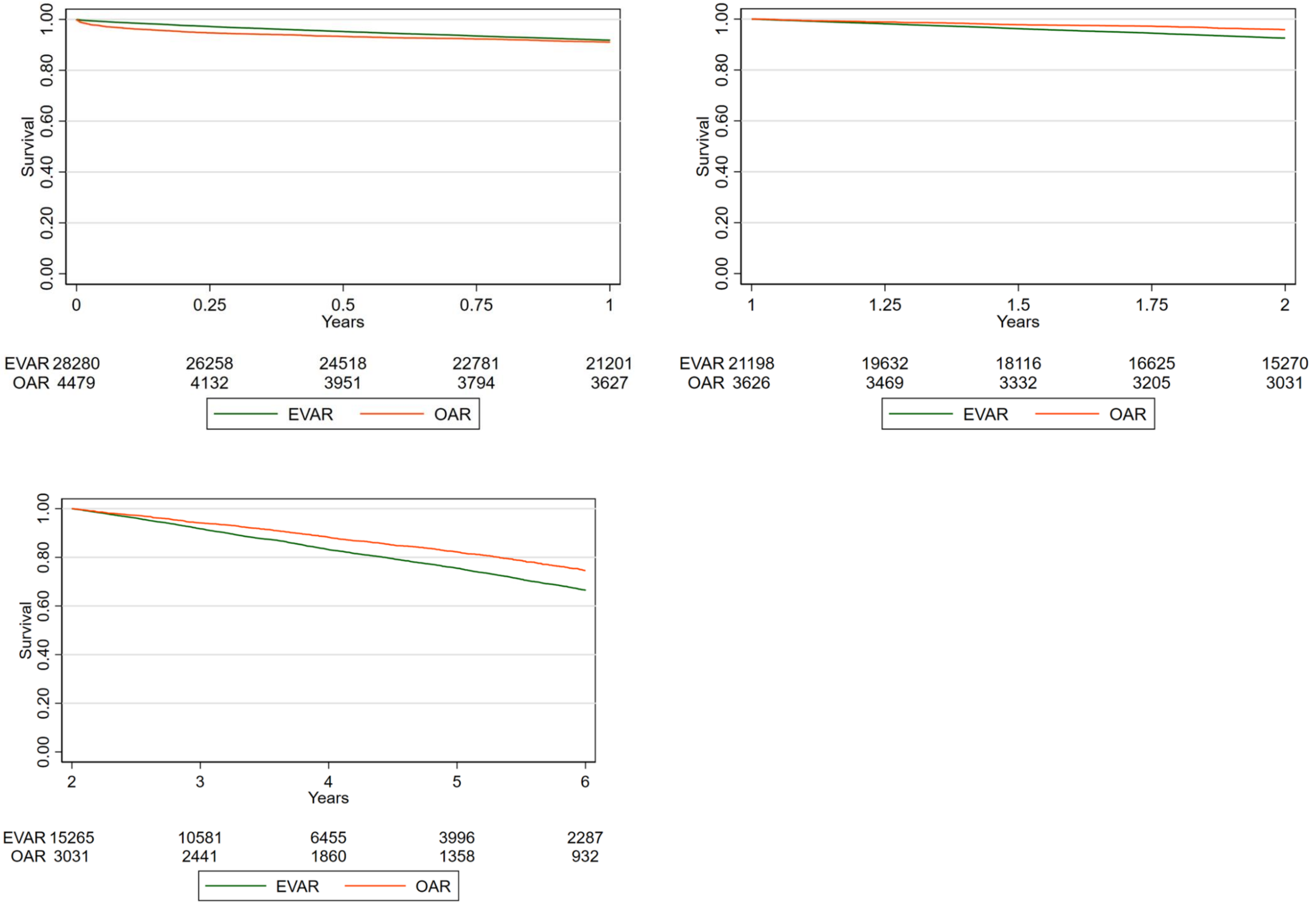

© 2022 Yei K et al. *JAMA Network Open.*

eFigure 3. Unmatched Mortality, Rupture, and Reintervention After AAA Repair

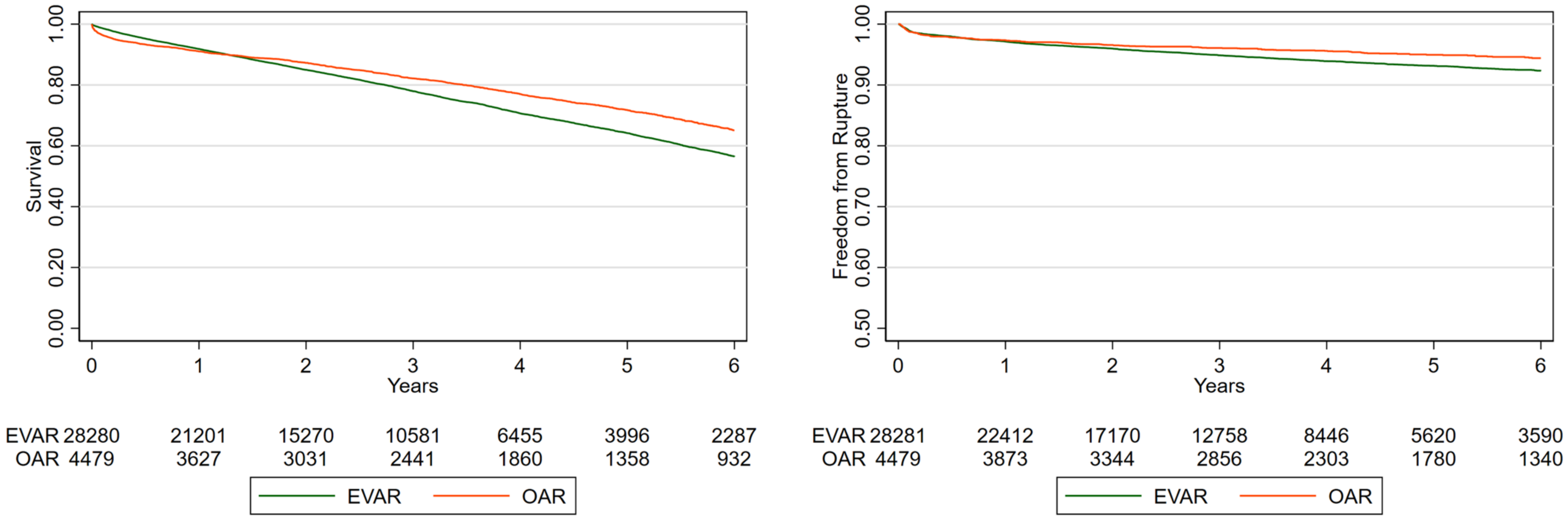

© 2022 Yei K et al. *JAMA Network Open.*

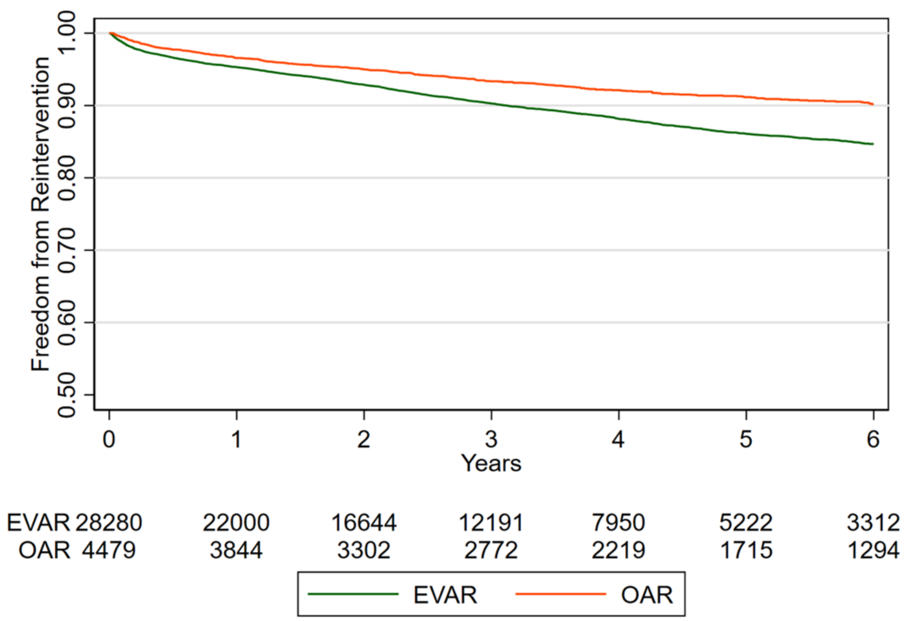

© 2022 Yei K et al. *JAMA Network Open.*
